# Beyond Meat: A Comparison of the Dietary Intakes of Vegetarian and Non-vegetarian Adolescents

**DOI:** 10.3389/fnut.2019.00086

**Published:** 2019-06-13

**Authors:** Gina Segovia-Siapco, Nasira Burkholder-Cooley, Sara Haddad Tabrizi, Joan Sabaté

**Affiliations:** ^1^Center for Nutrition, Healthy Lifestyle and Disease Prevention, School of Public Health, Loma Linda University, Loma Linda, CA, United States; ^2^Food Science and Nutrition Program, Chapman University, Orange, CA, United States

**Keywords:** vegetarian, adolescence, dietary habits, nutritional status, Adventist Health Studies, plant-based diets, NHANES, Adventist

## Abstract

Dietary intake of adult vegetarians from large prospective studies has been well-characterized but is rarely reported in vegetarian adolescents. Our objective was to describe and compare the dietary intake of vegetarian adolescents with their non-vegetarian counterparts in a population known to espouse healthy living. Adolescents (*n* = 534) aged 12–18 years old from middle and high schools near major Adventist universities in Michigan and Southern California provided dietary, demographic, and anthropometric data. Dietary intake was measured with a validated 151-item self-administered web-based food frequency questionnaire; weight and height were measured during school visits. Vegetarian was defined as the combined intake of meat, meat derivatives, poultry, and fish of <1 serving per week. Descriptive statistics and ANCOVA were used to compare the intake of vegetarians and non-vegetarians. Vegetarians significantly ate more fruits, vegetables, and other plant-based foods, but significantly less foods of animal origin, sugar-sweetened beverages, and coffee/tea compared to non-vegetarians. Vegetarians had significantly higher intakes of carbohydrates and total protein but lower intakes of fats, animal protein, and zinc compared to their counterparts. A majority (75% or more) of both groups met the 2015 Dietary Guidelines' age-and-gender-specific recommendations for most nutrients but only 16–18% of vegetarians/non-vegetarians did not exceed the upper limit for sodium. More vegetarians (49%) than non-vegetarians (25%) had <10% of their caloric intake from SFA. More than 90% of both groups met dairy recommendations, but greater proportions of vegetarians met recommendations for vegetables, fruits, nuts/soy products, and legumes than non-vegetarians. Of the non-vegetarians, only 7% and 44% met the fish and meats/poultry/eggs recommendation, respectively, which none of the vegetarians met. Compared to the general US adolescent population, both diet groups ate more fruits, vegetables, dairy and protein foods, and also consumed more micronutrients but less macronutrients. Overall, vegetarian adolescents have a more favorable dietary intake profile than non-vegetarians, but both vegetarians and non-vegetarians in this study population have a more adequate diet than the general US adolescent population. The influence of the Adventist plant-based diet culture that is translated both at home and at school is evident in our findings.

## Introduction

Dietary habits formed during childhood and adolescence have both short- and long-term impacts on health (1–3). Nutrient and energy demands increase to support the accelerated growth and development during adolescence, thus, it is crucial to establish or maintain healthy dietary habits during this period since this tends to persist through adulthood (4). A healthy diet during adolescence predisposes to better physical health since it is associated with lower weight gain (1) and lower risk of developing cardiovascular risk factors (5) during adulthood. High diet quality is also imperative to prevent depression (6), an increasing mental health problem among the youth and young adults (6–8).

Increased independence during adolescence and other factors, such as the family's socio-economic condition and education of parents (9), may influence dietary habits. Increased portion sizes of marketplace foods (10) have contributed to increased caloric consumption (11, 12) and low diet quality among US adolescents (13). Although there has been a decrease in sugar- sweetened beverages intake since 1999 (14), high-calorie-low nutrient-density foods have remained major sources of calories among the youth (12–14). On the contrary, intake of nutrient-dense foods (i.e., fruits and vegetables), remains below recommendations (15–18).

With a growing trend toward plant-based diets, there is an increasing movement toward vegetarian eating in different population groups, including adolescents. A vegetarian diet excludes meat, seafood, and products containing both, but may include eggs and dairy. Vegan diets eliminate all animal products. A national online survey conducted by Harris Poll for the Vegetarian Resource Group in 2014 indicated that 4% of youth ages 8–18 years are vegetarians while a total of 32% eat at least one vegetarian meal per week (19). In a 2016 national poll conducted in the United States among a representative sample of adults ages 18 years and above, 3.3% were self-reported vegetarians, half of whom claimed to be vegans (20).

Vegetarian and vegan diets are associated with a lower risk of chronic diseases among adults (21, 22). However, while plant-based or vegetarian diets and their health effects are well studied in adults, reports on vegetarian children and adolescents are scarce. We have access to the Adventist population, a group of people known to espouse a healthy lifestyle that includes a plant-based diet. A sizable proportion of this population are vegetarians, 4.2% being vegan and ~32% being lacto-ovo vegetarian (23). Although several studies have been published on Adventist adults, only very few have been reported on young people and none of these investigated their dietary intakes. Thus, our objectives were to describe and compare the dietary intake of Adventist vegetarian and non-vegetarian adolescents, determine if their reported intakes meet the recommend intakes for their age group, and compare their dietary profile with those of the general US adolescent population.

## Methods

### Study Design and Participants

In previous reports, we have described a cross-sectional study—the Teen Food and Development Study—that we conducted among adolescents to examine associations between dietary intake and certain health outcomes, particularly physical growth and pubertal development (24–26). We used the dietary data from this study in this report. Briefly, we collected data on 601 adolescents (262 males and 339 females) aged 12–18 years old from selected Adventist and public middle and high schools near major Adventist universities in Michigan and Southern California. A website was especially created for the study that served as the interface in the enrolment of potential participants and their parents, completing informed consent, and providing information on demographics, lifestyle habits, physical development, and dietary intake using the web-survey. Anthropometrics were measured during school visits.

Parents of potential participants 17 years old and younger provided their consent for their children's participation in the study by checking a box and providing their name and at least one telephone number at which they could be reached. In the same manner, the children indicated their assent to be part of the study by checking the assent box and providing their name on the same consent form. To ascertain that a parent actually provided the consent, a call was made at the telephone number provided. The informed consent was made official and printed for record-keeping only after the parent had confirmed his/her identity and consent, and the child's assent to participate. All study protocols, including data collection and informed consent, were approved by the Institutional Review Boards of Andrews University in Michigan (IRB#12-113) and Loma Linda University in Southern California (IRB#5120014).

### Assessment of Dietary Intake

Dietary intake was measured using a validated 151-item self-administered semi-quantitative web-based food frequency questionnaire designed for the study (27). Briefly, the questionnaire included 8 food groups: convenience foods (32 items), protein-rich foods (29 items), starches/cereals (17 items), vegetables/fruits (21 items), dairy products (10 items), beverages (24 items), snacks/sweets (11 items), and soups/legumes (7 items). Participants reported frequency of their intake of a food item using a drop-down list: never/rarely, 1–3 times per month, once per week, 2–4 times per week, 5–6 times per week, once per day, 2–3 times per day, and 4 or more times per day.

### Assessment of Vegetarian Status

Vegetarian status was determined based on self-reported intake of foods that differentiate vegetarians from non-vegetarians. Vegetarian was defined as intake of less than one combined portion (<3 ounces) of meat, meat derivatives, poultry, and fish per week.

### Assessment of Other Measures

During school visits, trained personnel measured weight and height of the participants. In the web-based survey, participants also self-reported their weight and height after measuring themselves. Clinical data for the anthropometrics were used for the statistical analysis; however, for participants who were absent during the school visits (about 4%), self-reported measures were used in the analysis since self-reported weight and height were found to be highly correlated to the clinic-measured values (28).

A section of the self-administered web survey included items on demographics and lifestyle (time spent on vigorous physical activity and sleep). The parents' educational levels were used as surrogate for socio-economic status. Some of these variables were controlled for when dietary intake comparisons were made between vegetarians and non-vegetarians.

### Data Management and Analysis

Of the 601 original participants, 13 were excluded due to completely missing diet or demographic data and 54 were excluded due to implausible energy intake (<500 calories and >3,500 calories for girls, <800 calories and >4,500 calories for boys). The final analytical dataset included 534 participants.

Descriptive and univariate analyses for all participants and by dietary preference (vegetarian vs. non-vegetarian) were conducted using either independent *t*-test or Chi-square test to check differences between vegetarians and non-vegetarians. ANCOVA was used to compare the food and nutrient intake of vegetarians and non-vegetarians while controlling for age, sex, ethnicity, education of mother, education of father, total energy intake, BMI z-score, and physical activity level. Before analysis, the food and nutrient variables were log-transformed due to non-normal distributions.

### Food Groupings and Nutrient Calculations

Foods were grouped according to the following categories: (1) breads, grains and pastas; (2) cereals; (3) fresh fruits; (4) 100% fruit juices, canned and dried fruits; (5) non-starchy vegetables; (6) starchy vegetables; (7) legumes; (8) meats (red meats, processed meats, and poultry); (9) fish; (10) meat alternatives; (11) nuts and nut butters; (12) eggs; (13) dairy cheese; (14) dairy milk; (15) dairy substitutes; (16) dairy desserts; (17) water; (18) sugar-sweetened beverages; (19) coffee and tea; and, (20) pastries and chips. The first 16 food groups were further re-categorized into breads/grains/pastas/cereals, total fruits, total vegetables, total protein foods, total dairy. Supplementary Table 1 shows the list of foods under each of the food groups. Given that our webFFQ was semi-quantitative, i.e., has fixed serving sizes, intake of these food groups was determined based on the sum of the intake frequency per day of the foods under each group, where frequency per day is equivalent to serving per day.

The nutrient profile of each food in the FFQ was determined using the Nutrition Data System for Research (29) database. Nutrient intake per day was computed using the sum-product method, i.e.,

(1) $$\begin{array}{r} A=\overset{n}{\underset{i=1}{\sum }}F_{i}a_{i} \end{array}$$

where *A* = total intake of nutrient *A* per day; F_*i*_ = frequency of food *i* intake per day; and a_*i*_ = amount of nutrient *A* in food *i*. The nutrients assessed in the study were: energy, total carbohydrates, added sugar, total fat, saturated fatty acids (SFA), mono-unsaturated fatty acids (MUFA), polyunsaturated fatty acids (PUFA), linoleic acid (LA), alpha linolenic acid (ALA), total protein, animal protein, vegetable protein, total dietary fiber, insoluble fiber, soluble fiber, vitamin B12, vitamin C, thiamin, riboflavin, vitamin D, vitamin E, folate, calcium, iron, potassium, magnesium, sodium, and zinc.

### Intake Comparisons With the US Adolescent Population

Food intakes of the study population were compared with their counterparts in the general US population using the values for those aged 14–18 years old in the 2007–2010 published estimates for the US population in the National Cancer Institute's Epidemiology and Genomics Research Program website (30). Weighted mean intake values for selected foods were computed since mean intakes were reported separately for males (*n* = 808) and females (*n* = 727). Percent differences between the mean intakes of the vegetarians and non-vegetarians in the study population were then calculated against the weighted means of the US adolescent population.

Nutrient intakes of the study population were also compared with the general US population ages 12–19 years using What We Eat in America, NHANES 2011–2012 (31) values from the Nutrient Intakes from Food and Beverages table. Since mean intake values were separated for male and female adolescents, weighted means were computed to represent the whole group. In the same manner as for foods, percent differences between the mean intakes of the vegetarians and non-vegetarians were then calculated against the general US population/NHANES values.

### Intake Comparisons With Dietary Guidelines and Dietary Reference Intakes

The US Dietary Guidelines Recommendations and Nutritional Goals for Age-Sex Groups Based on Dietary Reference Intakes and Dietary Guidelines Recommendations found in Appendix 7 of the 2015–2020 Dietary Guidelines for Americans (2015DGA) (32) was used to determine what proportions of the study population met the recommended intake amounts for selected nutrients. To determine if the participants met the recommended food groups intake, Healthy U.S.-Style Eating Pattern: Recommended Amounts of Food from Each Food Group at 12 Calorie Levels found in Appendix 3 of the 2015DGA was used. Comparisons were made according to age and gender group recommendations for nutrients and calorie levels of 1,600 kcal (for female participants ages 12–13 years old), 1,800 kcal (for male participants ages 12–13 years old and female participants ages 14–18 years old), and 2,200 kcal (for male participants ages 14–18 years old).

## Results

The demographic profile of the study population is presented in Table 1 for both vegetarians and non-vegetarians. Girls were more likely to be vegetarians compared to boys and more than half (52%) of the vegetarians were non-Hispanic whites. Although both groups had parents with college education or higher, the vegetarians had a significantly greater proportion of parents with higher education, especially at the graduate level. Vegetarians were also leaner compared to non-vegetarians. Non-vegetarians, however, spent significantly more time in vigorous physical activity compared to their vegetarian counterparts.

**Table 1:** Demographic characteristics according to vegetarian status.

**Variable**	**Non-vegetarians**, ***n*** **=** **397**		**Vegetarians**, ***n*** **=** **137**		***p*****-value**	
	***n* (%)**	**Mean (*SD*)**	***n* (%)**	**Mean (*SD*)**		
Sex						0.012
Girls	212 (53.4)		90 (65.7)			
Boys	185 (46.6)		47 (34.3)			
Age, years		15.0 (1.8)		15.0 (1.7)	0.986	0.458
12	28 (7.1)		10 (7.3)			
13	75 (18.9)		18 (13.1)			
14	64 (16.1)		25 (18.2)			
15	66 (16.6)		32 (23.4)			
16	70 (17.6)		25 (18.2)			
17	63 (15.9)		16 (11.7)			
18	31 (7.9)		11 (8.0)			
Mother's educational level						0.008
High school and below	66 (7.6)		8 (6.1)			
Vocational	18 (4.8)		5 (3.8)			
College	161 (42.9)		57 (43.2)			
Master's	101 (26.9)		42 (31.8)			
Doctoral	29 (7.7)		20 (15.2)			
Father's educational level						<0.001
High school and below	84 (22.4)		10 (7.6)			
Vocational	20 (5.3)		4 (3.0)			
College	128 (34.1)		34 (25.8)			
Master's	86 (22.9)		48 (36.4)			
Doctoral	57 (15.2)		36 (27.3)			
Ethnicity						0.001
Black	34 (9.1)		13 (9.8)			
Non-Hispanic White	123 (32.8)		69 (52.3)			
Hispanic	60 (16.0)		13 (9.8)			
Asian	49 (13.1)		13 (9.8)			
Other ethnicities	109 (29.1)		24 (18.2)			
Site						0.325
California	225 (56.7)		71 (51.8)			
Michigan	172 (43.3)		66 (48.2)			
Physical activity, min/day		33.3 (25.7)		27.5 (23.1)	0.022	
Duration of sleep, hours/day		7.8 (2.5)		7.9 (1.1)	0.746	
BMI Z-score		0.42 (0.97)	0.14 (0.89)		0.005	

As shown in Table 2, there were significant differences in the food intakes of vegetarians and non-vegetarians. Table 2 displays the estimated marginal means with their 95% confidence intervals after controlling for relevant covariates. Vegetarians ate about one-half serving more of fruits, nearly serving more of vegetables, and over one serving more of nut/nut butters and meat alternatives than their non-vegetarian peers. Not surprisingly, non-vegetarians ate slightly over one serving more of animal protein foods than the non-vegetarians, but their egg intake amounted to approximately one-third of a serving compared to about one-fourth of a serving for their vegetarian counterparts. Intake of dairy products by non-vegetarians was higher by just a small amount (between 0.07 to 0.30 of a serving) compared to vegetarians. Although intakes of sugar-sweetened beverages and coffee/tea were small for both groups (much less than a serving), the non-vegetarians significantly consumed more of these beverages than the vegetarians.

**Table 2:** Comparison of the intake of selected foods by diet groups.

**Foods, servings per day**	**Means (95% CI)^a^**		***p*-value**
	**Non-vegetarians**	**Vegetarians**	
Breads/Grains/Pastas/Cereals, total	5.42 (5.25, 5.59)	**5.82 (5.53, 6.13)**	0.022
Breads, Grains, Pastas	4.75 (4.60, 4.91)	4.97 (4.69, 5.26)	0.207
Cereals	0.58 (0.53, 0.63)	**0.75 (0.65, 0.86)**	0.003
Fruits, total	2.17 (2.03, 2.31)	**2.71 (2.44, 3.00)**	0.001
Fresh fruits	1.53 (1.42, 1.65)	**1.93 (1.70, 2.17)**	0.002
100% FJ; canned and dried fruits	0.60 (0.54, 0.66)	0.68 (0.58, 0.79)	0.177
Vegetables, total	3.54 (3.37, 3.71)	**4.32 (3.99, 4.67)**	<0.0001
Non-starchy vegetables	2.66 (2.52, 2.81)	**3.11 (2.85, 3.40)**	0.004
Starchy vegetables	0.30 (0.27, 0.32)	0.30 (0.26, 0.34)	0.958
Legumes	0.47 (0.43, 0.52)	**0.76 (0.68, 0.85)**	<0.0001
Protein foods, total	2.44 (2.35, 2.54)	2.30 (2.15, 2.46)	0.144
Meat, poultry, eggs	**1.37 (1.30, 1.44)**	0.29 (0.22, 0.36)	<0.0001
Meats (red meats, poultry)	**0.97 (0.92, 1.03)**	0.06 (0.01, 0.11)	<0.0001
Eggs	**0.37 (0.34, 0.40)**	0.23 (0.18, 0.28)	<0.0001
Fish	**0.10 (0.09, 0.12)**	0.01 (-0.01, 0.03)	<0.0001
Nuts, nut butters, meat alternatives	0.86 (0.79, 0.93)	**1.97 (1.77, 2.17)**	<0.0001
Meat alternatives	0.55 (0.49, 0.61)	**1.34 (1.19, 1.50)**	<0.0001
Nuts and nut butters	0.29 (0.26, 0.33)	**0.53 (0.45, 0.61)**	<0.0001
Dairy, total	3.25 (3.16, 3.34)	3.08 (2.94, 3.22)	0.058
Cheese, dairy	**2.03 (2.01, 2.06)**	1.96 (1.92, 2.00)	0.003
Milk, dairy	**0.53 (0.47, 0.59)**	0.23 (0.15, 0.31)	<0.0001
Dairy desserts	**0.30 (0.27, 033)**	0.22 (018, 0.27)	0.007
Dairy substitutes	0.24 (0.19, 0.28)	**0.51 (0.42, 0.61)**	<0.0001
Water	2.75 (2.57, 2.95)	3.08 (2.74, 3.45)	0.118
Sugar-sweetened beverages	**0.62 (0.57, 0.69)**	0.41 (0.32, 0.50)	<0.0001
Coffee and tea	**0.17 (0.14, 0.20)**	0.09 (0.04, 0.13)	0.002
Pastries and chips	1.10 (1.02, 1.17)	1.10 (0.97, 1.24)	0.938

a *Estimated marginal means; controlled for age, gender, ethnicity, education of mother, education of father, total energy intake, BMI z-scores, and physical activity*.

*Values in bold are significantly greater than the other diet group*.

Table 3 shows a comparison of the nutrient intakes between the two diet groups. Vegetarians had significantly higher intakes of carbohydrates (~27 g more), polyunsaturated fatty acids (~3 g more PUFA and LA, and ~2 g more ALA), LA:ALA ratio, vegetable protein (~21 g more), dietary fiber (~8 g more), vitamin C (~20 mg more), thiamin (~1.7 mg more), vitamin E (~1.4 mg more), folate (~135 mcg more), calcium (~130 mg more), iron (3.6 mg more), potassium (~296 mg more), magnesium (~72 mg more), as well as sodium (~150 mg more) and phosphorus (57 mg more). On the other hand, non-vegetarians significantly consumed more total (~6 g more), saturated (~5 g more) and monounsaturated (~3 g more) fats, animal protein (~23 g more), and zinc (1 mg more) compared to vegetarians.

**Table 3:** Comparison of the intake of selected nutrients by vegetarians and non-vegetarians.

**Nutrients (servings per day)**	**Means (95% CI)^a^**		***p*-value**
	**Non-vegetarians**	**Vegetarians**	
Energy, kcal	1990.22 (1972.39, 2008.21)	2010.22 (1980.29, 2040.60)	0.277
Total carbohydrates, g	248.64 (244.69, 252.90)	**275.06 (267.20, 283.16)**	<0.0001
Added sugars	40.73 (38.74, 42.78)	37.15 (34.09, 40.49)	0.077
Fat	**77.09 (75.57, 78.65)**	70.95 (68.58, 73.48)	<0.0001
SFA, g	**26.68 (25.89, 27.47)**	21.41 (20.35, 22.53)	<0.0001
MUFA, g	**25.97 (25.41, 26.52)**	22.71 (21.89, 23.59)	<0.0001
PUFA, g	17.41 (16.98, 17.85)	**20.78 (19.91, 21.69)**	<0.0001
LA	15.27 (14.89, 15.67)	**18.38 (17.58, 19.20)**	<0.0001
ALA	1.70 (1.66, 1.74)	**1.86 (1.78, 1.93)**	<0.0001
LA:ALA ratio	8.98 (8.83, 9.14)	**9.90 (9.60, 10.22)**	<0.0001
Total protein, g	79.84 (78.34, 81.37)	77.48 (74.89, 80.08)	0.124
Animal protein	**38.21 (36.60, 39.92)**	15.72 (14.57, 16.96)	<0.0001
Vegetable protein, g	37.68 (36.45, 38.98)	**58.62 (55.37, 62.12**)	<0.0001
Total dietary fiber, g	21.87 (21.24, 22.49)	**29.84 (28.42, 31.37)**	<0.0001
Insoluble fiber, g	15.35 (14.88, 15.82)	**21.31 (20.19, 22.47)**	<0.0001
Soluble fiber, g	6.45 (6.28, 6.62)	**8.46 (8.08, 8.86)**	<0.0001
Vitamin B12. ug	5.69 (5.44, 5.95)	5.85 (5.42, 6.32)	0.539
Vitamin C, mg	142.74 (134.96, 150.96)	**162.39 (147.23, 178.93)**	0.029
Thiamin, mg	2.32 (2.20, 2.44)	**4.03 (3.69, 4.42)**	<0.0001
Riboflavin, mg	2.03 (1.98, 2.08)	2.10 (2.01, 2.19)	0.174
Vitamin D, mcg	4.69 (4.42, 4.98)	4.18 (3.77, 4.64)	0.067
Vitamin E, mg α-tocopherol	8.46 (8.20, 8.73)	**9.84 (9.31, 10.39)**	<0.0001
Folate, ug	540.77 (524.79, 557.80)	**675.19 (640.34, 711.94)**	<0.0001
Calcium, mg	1091.16 (1059.97, 1122.15)	**1221.70 (1162.12, 1284.34)**	<0.0001
Iron, mg	16.58 (16.15, 17.03)	**20.19 (19.30, 21.14)**	<0.0001
Potassium, mg	2702.68 (2643.87, 2765.56)	**2998.90 (2884.19, 3118.17)**	<0.0001
Magnesium, mg	314.51 (308.28, 320.86)	**386.45 (373.16, 399.81)**	<0.0001
Sodium, mg	3248.67 (3190.71, 3310.98)	**3398.20 (3291.18, 3508.70)**	0.022
Zinc, mg	**11.93 (11.66, 12.19)**	10.92 (10.52, 11.36)	<0.0001

a *Estimated marginal means; controlled for age, gender, ethnicity, education of mother, education of father, total energy intake (except for energy), BMI z-scores, and physical activity level*.

*Values in bold are significantly greater than the other diet group*.

As seen in Table 4, at least 75% of both vegetarians and non-vegetarians in the study population met the recommended intake amounts for carbohydrates, protein, vitamin B12, riboflavin, vitamin C, thiamin, folate, and iron, and had adequate intakes of the omega-3 fatty acid, alpha linolenic acid (ALA). About 77% of vegetarians reported adequate intake of linoleic acid compared to about 72% of non-vegetarians. Very small proportions (≤10%) of both vegetarians and non-vegetarians met the dietary recommendations for vitamins D and E and potassium, and <20% consumed sodium below the upper limit. Almost 50% of the vegetarians consumed <10% of their total caloric intake from saturated fatty acids (SFA) whereas only half of that proportion (~25%) met the SFA intake recommendation among non-vegetarians. Compared to non-vegetarians, a larger proportion of vegetarians met the dietary fiber (53 vs. 36%), calcium (44 vs. 36%), and magnesium (56 vs. 48%) recommendations. On the other hand, a larger proportion of non-vegetarians met the zinc recommendations compared to their vegetarian counterparts (77 vs. 64%).

**Table 4:** Percentage who met age-and-gender specific recommendations for intake of selected nutrients and food groups.

	**Met Recommendations, %**		
	**All subjects**	**Non-vegetarians**	**Vegetarians**
NUTRIENTS^a^			
Carbohydrates	95.1	94.7	96.4
Protein	92.5	93.2	90.5
SFA <10% of energy intake	31.1	24.9	48.9
Linoleic acid (LA)^c^	73.0	71.5	77.4
Linolenic acid (ALA)^c^	77.7	77.6	78.1
Dietary fiber	40.6	36.3	53.3
Vitamin B12	92.7	94.5	87.6
Riboflavin	93.8	94.2	92.7
Vitamin C	88.8	88.4	89.8
Thiamin	94.6	92.9	99.3
Folate	82.0	80.1	87.6
Vitamin D	3.4	3.3	3.6
Vitamin E	15.4	15.1	16.1
Calcium	37.6	35.5	43.8
Iron	78.5	76.8	83.2
Potassium^c^	9.0	8.6	10.2
Magnesium	50.0	48.1	55.5
Sodium^d^	17.6	18.1	16.1
Zinc	73.6	76.8	64.2
Phosphorus	57.7	58.2	56.2
FOOD GROUPS^b^			
Vegetables^e^	71.2	68.0	80.3
Starchy vegetables^f^	7.9	8.3	6.6
Non-starchy vegetables^f^	78.7	76.6	84.7
Legumes^f^	77.3	73.6	88.3
Fruits^e^	64.8	62.5	71.5
Grains, oz-eq/day	40.4	41.3	38.0
Dairy^e^	95.1	95.5	93.8
Protein foods^g^	55.4	61.0	39.4
Meats/poultry/eggs^h^	33.0	44.3	0.0
Fish^h^	5.2	7.1	0.0
Nuts and soy products^h^	77.3	70.3	97.8

a *Intake of study participants were compared with Appendix 7 Nutritional Goals for Age-Sex Groups Based on Dietary Reference Intake and Dietary Guidelines Recommendations (27)*.

b *Intake of study participants were compared with Table A3-1 Healthy U.S.-Style Eating Pattern: Recommended Amounts of Food from Each Food Group at 12 Calorie Levels(27)*.

c *Based on adequate intake (AI) goal*.

d *Based on upper limit (UL) goal*.

e *Based on cup-equivalents per day*.

f *Measured in cup-equivalents per week*.

g *Measured in oz-equivalents per day*.

h *Measured in oz-equivalents per week*.

Table 4 also shows the percentage of study participants who met the recommendations for vegetables, fruits, grains, dairy, and protein foods. Compared to their non-vegetarian counterparts, a larger percentage of vegetarians ate more vegetables (80 vs. 68%), non-starchy vegetables (85 vs. 77%), legumes (88 vs. 74%), fruits (72 vs. 62%), and nuts/soy products (98 vs. 70%). Only a small proportion of both groups met the recommendations for starchy vegetables, while more non-vegetarians met the recommendations for grains (41 vs. 38%) and protein foods (61 vs. 39%) than vegetarians. No vegetarian participants met the meat/poultry/eggs and fish intake recommendations.

**Figure 1:**
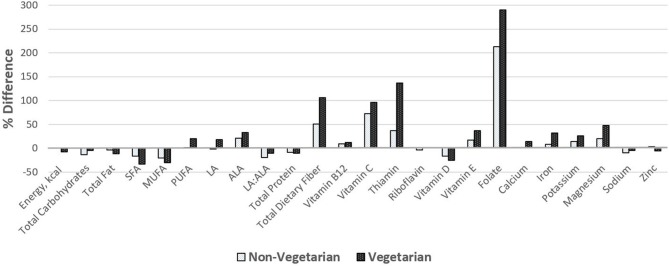
Percent difference in food group intake of vegetarian and non-vegetarian participants compared to the average intake of the US adolescent population. Comparisons were made using data from *Usual Dietary Intakes: Food Intakes, US Population, 2007–2010* (30). Bars above the reference “0” line indicate higher intake while bars below the line indicate lower intake compared to the average intake of US adolescents.

**Figure 2:**
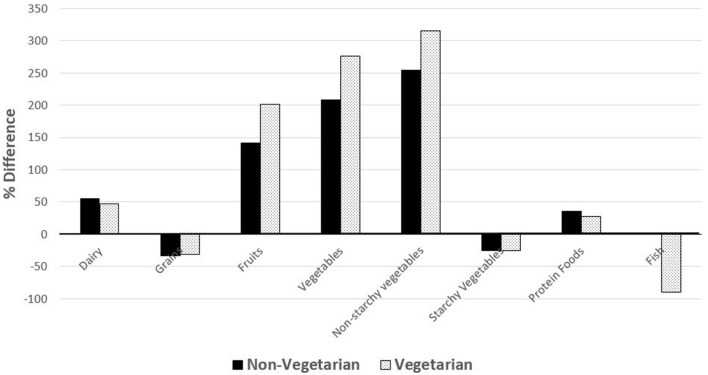
Percent difference in nutrient intake of vegetarian and non-vegetarian participants compared to the average US adolescent population intake. Comparisons were made with nutrient intakes of adolescents from *What We Eat in America*, NHANES 2011–2012 for US population ages 12–19 years (31). Bars above the 0 horizontal line means greater intake while bars below the 0 horizontal line means lesser intake for participants compared to the reported intakes of the reference US adolescent population.

Figures 1, 2 show comparisons of percent intake differences for food groups (Figure 1) and nutrients (Figure 2) between the two diet groups and the general US adolescent population. Intakes of dairy, fruits, total vegetables, non-starchy vegetables, and protein foods were relatively greater (ranging from about 25% to 300% more), whereas intakes of breads/grains/pastas, starchy vegetables, and fish were relatively lower (ranging from about 20 to 90%) for both diet groups than average US adolescents. However, only vegetarians reported lower intake of fish while non-vegetarians had a fish intake similar to that of US adolescents. Intakes of ALA, dietary fiber, vitamin B12, vitamin C, thiamin, vitamin E, folate, iron, potassium, and magnesium were higher for both groups (ranging from ~10 to ~290%) except for zinc, which was only higher for the non-vegetarian group compared to the US adolescent population. Only vegetarians had higher intakes of polyunsaturated fatty acids (PUFA), linoleic acid (LA), and calcium. Intakes of total fat, SFA, monounsaturated fatty acids (MUFA), LA:ALA ratio, total protein, vitamin D, and sodium were lower for both groups compared to the US adolescents, but zinc intake was only lower for vegetarians.

## Discussion

In this study, we described and compared the dietary intake of vegetarian and non-vegetarian adolescents in a population largely composed of Adventists. We further compared these intakes with the DRI and 2015 DGA to determine dietary intake adequacy, and with those of the general US adolescent population. As expected, vegetarians significantly consumed more plant-based foods (i.e., fruits, vegetables, legumes, cereals), meat alternatives, and dairy substitutes compared to their non-vegetarian counterparts while the non-vegetarians consumed more dairy and protein foods from animal sources (i.e., meats, eggs, and fish). Vegetarians also consumed higher amounts of most nutrients except those that are associated with animal foods (total fat, SFA, animal proteins, and zinc) and MUFA compared to non-vegetarians. However, in comparison to the reported intake of US adolescents, both diet groups in our study population ate more plant-based foods (fruits, vegetables), dairy, and protein foods but relatively less breads/grains/pastas, starchy vegetables, and fish. Regarding nutrients, both groups had similar intakes of total energy, added sugars, total protein, vitamin B12, riboflavin, and vitamin D. Compared to US adolescents, both diet groups have lower intakes of total energy and macronutrients except for PUFA, ALA and dietary fiber; however, intake of LA was only higher for vegetarians but similar for non-vegetarians. Intakes of nutrients associated with fruits and vegetables (e.g., folate and vitamin C) and nuts/nut butters (e.g., vitamin E and ALA) as well as most of the selected micronutrients, except vitamin D and sodium, were higher for both groups compared to their US adolescent counterparts. However, zinc intake was higher for non-vegetarians yet lower for vegetarians compared to US adolescents. Most of the nutrient recommendations were met by more than three-fourths of both vegetarians and non-vegetarians except for linoleic acid and zinc. About half of vegetarians but just 25% of non-vegetarians met the recommendation for SFA intake to be <10% of total energy intake, while over half of vegetarians and about one-third of non-vegetarians met the dietary fiber recommendation. Lower proportions of both groups met the recommendations for intake of the nutrients important for bone health: calcium (44 vs. 36%, vegetarians vs., non-vegetarians, respectively); magnesium (56 vs. 48%); and, vitamin D (3.6 vs. 3.3%).

Our findings are consistent with previous findings of Perry et al. (33) regarding intakes of fruits, vegetables, total and saturated fats, and iron among vegetarian and non-vegetarian adolescents. However, unlike their findings, vegetarians in our study had similar vitamin B12 intake as non-vegetarians, probably due to most of them being lacto-ovo vegetarians. Vitamin D intake in both vegetarians and non-vegetarians is lower in this adolescent group compared to the mean intake (5.6 mcg) of the NHANES 2011–2012 adolescent population (31). In the NHANES 2007–2010, it was reported that 88% of US youth ages 2–18 did not meet the estimated average requirement (EAR) for vitamin D (34). When compared with other populations, the mean intake of vitamin D is also lower in our population than that of adolescent Finnish vegans (5 mcg) and omnivores (14 mcg) (35). As a vitamin involved in bone metabolism, this is a concern; however, a better measure of the vitamin D status is serum 25(OH)D, which should be measured in this population to assess vitamin D adequacy.

The LA:ALA ratios for our study population-−8.98 for non-vegetarians and 9.90 for vegetarians—are lower compared to the US adolescent population (weighted mean = 11.1). A lower LA:ALA ratio is considered favorable since the dietary ratio of LA (the parent n-6) to ALA (the parent n-3) had been shown to affect ALA conversion to the physiologically important very long-chain n-3 fatty acids, EPA and DHA, in the red blood cells (36, 37). A lower LA intake and a LA:ALA ratio <10 may improve this conversion and also reduce arachidonic acid (37), the precursor of pro-inflammatory eicosanoids.

The majority of both diet groups (82% of non-vegetarians and 84% of vegetarians) have exceeded the recommended upper limit (2,200 mg for ages 9–13 and 2,300 mg for ages 14–18) of sodium; these proportions are slightly lower than the reported excessive sodium intake prevalence in the US (89% among adults and >90% among children) (38). This is alarming given that the average intakes for both groups exceeded sodium intake limits by about 1,000 mg. Excessive sodium intake had been consistently linked to negative health effects, specifically elevated blood pressure and risk of kidney disease (39). Upon further analysis using stepwise multiple regression, we found that bread/pasta/grains explained 59.4% of the total variance in sodium intake. This was followed by legumes, which contributed 14.1% to the variance, succeeded by dairy cheese and non-starchy vegetables (each contributed 6.5% of total variance), meat alternatives (3.8%), meats/poultry (2.0%), starchy chips/pastry (1.4%), and others that contributed <1% to the total sodium intake variance of 96.7%. Foods under these groupings are similar to what were identified as major sources of sodium intake among US children ages 6–18 years as reported in NHANES 2011–2012 (40) and NHANES 2003–2008 (41), most of which are either manufactured/processed or from fast foods. Also consistent with the NHANES 2005–2008 report (42), we found sugar-sweetened beverages to be a significant predictor of sodium intake, but it only explained 0.1% of the total variance.

Using the same stepwise algorithm, we explored further the main sources of variation in SFA intake in our study population and found dairy cheese to be the main contributor (48.3% of the 72.5% total variance). Other foods that are significant contributors were meats and chicken (9.3%), dairy desserts (6.3%), chips and pastries (4.4%), milk/yogurt (2.1%), non-starchy vegetables (1.0%), and <1% each from eggs, nuts/nut butters, and starchy vegetables. The top five sources of SFA intake among US adolescents based on the NHANES 2011–2014 data were pizza, sweet bakery products, milk, Mexican dishes, and cheese (43). Cheese intake in our population is correlated with intake of pizza aside from other foods, such as pasta and sandwiches. Meats, poultry, cured meats/poultry and mixed dishes containing meat/fish/poultry were ranked 9th, 10th, 12th, and 15th, respectively, as sources of SFA among US adolescents (43), whereas meats and chicken were ranked second as a source of SFA in our population. Again, it should be noted that most foods identified to be determinants of saturated fat intakes are also manufactured/processed or from fast foods. In light of these findings, studies are needed to determine the impact of excessive salt intake during adolescence, both among vegetarians and non-vegetarians, as well as saturated fat intake >10% of total caloric intake, on health during adulthood.

Many other studies on vegetarian adolescents compared dietary intakes for only a few foods or nutrients (44), or were conducted on younger non-US populations (45–47). Our study is one of the very few that investigated the dietary intake of vegetarian adolescents. One strength of our study is the multi-ethnicity and the large percentage of vegetarians (25%) that provided the power necessary to determine significant intake differences between the diet groups. However, since our study population may not be representative of the US adolescent population, this limits the generalizability of our findings. On the other hand, our findings were also consistent with those of the previous study (33), despite methodological differences, and thus add additional evidence that vegetarian adolescents tend to have a more favorable dietary intake profile. One limitation of our study is the use of a dietary assessment method that has inherent biases, including the possibility of over- or under-estimation of reported intakes. However, food frequency questionnaires continue to be the mainstay in assessing habitual diet because they are low-cost, time-efficient, and easy to administer especially in the context of large epidemiological studies. The lack of biomarkers used to verify nutrient status based on reported intake is another limitation. Currently, however, only a few valid and reliable nutritional biomarkers have been identified that can be feasibly used in population studies (48). Considering that a substantial number of 9–13 (30%) and 14–18 (26.3%) years old US children use dietary supplements (49), we may have underestimated the nutrient intake of our participants by not accounting for their supplement use. However, our intent was to a focus on the nutrient amounts that our adolescent population derive from their food intake. The comparison values from the reference US adolescent population were also only based on nutrient intake from foods and beverages (31).

## Conclusions

Overall, the influence of the SDA plant-based diet culture that is translated both at home and at school is evident in our findings. We found vegetarian SDA adolescents to have a more favorable dietary intake profile than non-vegetarians as indicated by the higher intake of plant-based foods and lower intake of nutrients and foods associated with detrimental health effects, such as saturated fats and animal-based products. It is notable that both vegetarians and non-vegetarians in this study population ate healthier than the general US adolescent population. This is indicated by vegetarians' greater intakes of foods considered to be beneficial to health and their overall better nutrient intake profile, including lower intakes of energy and SFA, and higher intakes of dietary fiber and most nutrients of concern—folate, iron, and the bone minerals calcium, potassium, and magnesium. However, a large majority of both the vegetarians and non-vegetarians exceeded the upper intake limits for sodium just like the general US adolescents. Our study does not allow us to draw conclusions about the potential beneficial effects of a long-term vegetarian diet in adult life endpoints. Thus, we recommend further investigations of the dietary tracking from adolescence to adulthood and the impact of dietary intake during adolescence to future health.

## Data Availability

The datasets for this manuscript are not publicly available because we are still using the data for writing other manuscripts/reports. Requests to access the datasets should be directed to Joan Sabate, jsabate@llu.edu.

## Ethics Statement

This study was carried out in accordance with the recommendations of the Institutional Review Board of Loma Linda University with written informed consent from parents and assent of all subjects. All subjects gave written informed consent in accordance with the Declaration of Helsinki. The protocol was approved by the Institutional Review Boards of Loma Linda University and Andrews University.

## Author Contributions

JS and GS-S designed the study. GS-S supervised the data collection, analyzed the data, and prepared the manuscript. JS, NB-C, SH, and GS-S interpreted the data and critically reviewed, edited, and finalized the manuscript.

### Conflict of Interest Statement

The authors declare that the research was conducted in the absence of any commercial or financial relationships that could be construed as a potential conflict of interest.

## References

[1] Hu T Jacobs DR Larson NI Cutler GJ Laska MN Neumark-Sztainer D. Higher diet quality in adolescence and dietary improvements are related to less weight gain during the transition from adolescence to adulthood. J Pediatr US. (2016) 178:188–93.e3. 10.1016/j.jpeds.2016.08.026 27640354 PMC5085861

[2] Emmett PM Jones LR. Diet, growth, and obesity development throughout childhood in the Avon longitudinal study of parents and children. Nutr Rev. (2015) 73:175–206. 10.1093/nutrit/nuv054 26395342 PMC4586450

[3] Malik VS Fung TT van Dam RM Rimm EB Rosner B Hu FB. Dietary patterns during adolescence and risk of type 2 diabetes in middle-aged women. Diabetes Care. (2012) 35:12–8. 10.2337/dc11-0386 22074723 PMC3241320

[4] Movassagh EZ Baxter-Jones ADG Kontulainen S Whiting SJ Vatanparast H. Tracking dietary patterns over 20 years from childhood through adolescence into young adulthood: the Saskatchewan pediatric bone mineral accrual study. Nutrients. (2017) 9:990. 10.3390/nu9090990 28885565 PMC5622750

[5] Dahm CC Chomistek AK Jakobsen MU Mukamal KJ Eliassen AH Sesso HD. Adolescent diet quality and cardiovascular disease risk factors and incident cardiovascular disease in middle-aged women. J Am Heart Assoc. (2016) 5:e003853. 10.1161/JAHA.116.003583 27998915 PMC5210420

[6] Opie RS Itsiopoulos C Parletta N Sanchez-Villegas A Akbaraly TN Ruusunen A. Dietary recommendations for the prevention of depression. Nutr Neurosci. (2017) 20:161–71. 10.1179/1476830515Y.0000000043 26317148

[7] Mojtabai R Olfson M Han B. National trends in the prevalence and treatment of depression in adolescents and young adults. Pediatrics. (2016) 138:e20161878. 10.1542/peds.2016-1878 27940701 PMC5127071

[8] Banta JE Khoie-Mayer RN Somaiya CK McKinney O Segovia-Siapco G. Mental health and food consumption among California children 5-11 years of age. Nutr Health. (2013) 22:237–53. 10.1177/0260106015599511 26399270

[9] Harris C Flexeder C Thiering E Buyken A Berdel D Koletzko S. Changes in dietary intake during puberty and their determinants: results from the GINIplus birth cohort study. BMC Public Health. (2015) 15:841. 10.1186/s12889-015-2189-0 26329931 PMC4556194

[10] Young LR Nestle M. The contribution of expanding portion sizes to the US obesity epidemic. Am J Public Health. (2002) 92:246–9. 10.2105/AJPH.92.2.246 11818300 PMC1447051

[11] Poti JM Popkin BM. Trends in energy intake among US children by eating location and food source, 1977-2006. J Am Diet Assoc. (2011) 111:1156–64. 10.1016/j.jada.2011.05.007 21802561 PMC3148484

[12] Slining MM Mathias KC Popkin BM. Trends in food and beverage sources among US children and adolescents: 1989-2010. J Acad Nutr Diet. (2013) 113:1683–94. 10.1016/j.jand.2013.06.001 23916972 PMC3905608

[13] Murakami K Livingstone MB. Associations between meal and snack frequency and overweight and abdominal obesity in US children and adolescents from National Health and Nutrition Examination Survey (NHANES) 2003-2012. Br J Nutr. (2016) 115:1819–29. 10.1017/S0007114516000854 27001436

[14] Larson N Story M Eisenberg ME Neumark-Sztainer D. Secular trends in meal and snack patterns among adolescents from 1999 to 2010. J Acad Nutr Diet. (2016) 116:240–50e2. 10.1016/j.jand.2015.09.013 26482095 PMC4733410

[15] US Department of Agriculture All About the Fruit Group. (2016). Available online at: https://www.choosemyplate.gov/fruit

[16] US Department of Agriculture All About the Vegetable Group. (2016). Available online at https://www.choosemyplate.gov/vegetables

[17] Bradlee ML Singer MR Qureshi MM Moore LL. Food group intake and central obesity among children and adolescents in the Third National Health and Nutrition Examination Survey (NHANES III). Public Health Nutr. (2010) 13:797–805. 10.1017/S1368980009991546 19772691

[18] Herrick KA Rossen LM Nielsen SJ Branum AM Ogden CL. Fruit consumption by youth in the United States. Pediatrics. (2015) 136:664–71. 10.1542/peds.2015-1709 26391940 PMC4774519

[19] Vegetarian Resource Group The Vegetarian Resource Group Blog [Internet]. Baltimore, MD: The Vegetarian Resource Group (2014). (cited 2018). Available online at: https://www.vrg.org/blog/2014/05/30/how-many-teens-and-other-youth-are-vegetarian-and-vegan-the-vegetarian-resource-group-asks-in-a-2014-national-poll/

[20] Stahler C How Many Adults are Vegetarians? The Vegetarian Resource Group (2015). Available online at: https://www.vrg.org

[21] Key TJ Appleby PN Rosell MS. Health effects of vegetarian and vegan diets. Proc Nutr Soc. (2006) 65:35–41. 10.1079/PNS2005481 16441942

[22] Orlich MJ Fraser GE. Vegetarian diets in the Adventist Health Study 2: A review of initial published findings. Am J Clin Nutr. (2014) 100:353s−8s. 10.3945/ajcn.113.071233 24898223 PMC4144107

[23] Butler TL Fraser GE Beeson WL Knutsen SF Herring RP Chan J. Cohort profile: The Adventist Health Study-2 (AHS-2). Int J Epidemiol. (2008) 37:260–5. 10.1093/ije/dym165 17726038

[24] Segovia-Siapco G Pribis P Messina M Oda K Sabate J. Is soy intake related to age at onset of menarche? A cross-sectional study among adolescents with a wide range of soy food consumption. Nutr J. (2014) 13:54. 10.1186/1475-2891-13-54 24889551 PMC4051381

[25] Nezami M Segovia-Siapco G Beeson WL Sabate J. Associations between consumption of dairy foods and anthropometric indicators of health in adolescents. Nutrients. (2016) 8:427. 10.3390/nu8070427 27420094 PMC4963903

[26] Segovia-Siapco G Pribis P Oda K Sabate J. Soy isoflavone consumption and age at pubarche in adolescent males. Eur J Nutr. (2017) 57:2287–94. 10.1007/s00394-017-1504-1 28712053

[27] Segovia-Siapco G Oda K Sabaté J Evaluation of the relative validity of a Web-based food frequency questionnaire used to assess Soy Isoflavones and nutrient intake in adolescents. BMC Nutr. (2016) 2:39 10.1186/s40795-016-0080-8

[28] Siapco GS Alomairah S Sabate J Can teens accurately report their weight, height, and waist and hip circumferences? FASEB J. (2013) 27.

[29] Nutrition Coordinating Center University of Minnesota Nutrition Data System for Research (NDS-R) 2013 Minneapolis, MN: The Nutrition Coordinating Center, Division of Epidemiology, School of Public Health, University of Minnesota (2000).

[30] Epidemiology and Genomics Research Program Usual Dietary Intakes: Food Intakes, U.S. Population, 2007-10. National Cancer Institute (2018) (updated April 24, 2018). Available online at: https://epi.grants.cancer.gov/diet/usualintakes/pop/2007-10/

[31] US Department of Agriculture and Agricultural Research Service Nutrient Intakes from Food: Mean Amounts Consumed per Individual, by Gender and Age. What We Eat in America, NHANES 2011-2012 (2012). Available at online https://www.ars.usda.gov/northeast-area/beltsville-md-bhnrc/beltsville-human-nutrition-research-center/food-surveys-research-group/docs/wweia-data-tables/

[32] U.S. Department of Health and Human Services and U.S. Department of Agriculture. 2015–2020 Dietary Guidelines for Americans. 8th Edition (2015). Available online at http://health.gov/dietaryguidelines/2015/guidelines/

[33] Perry CL McGuire MT Neumark-Sztainer D Story M. Adolescent vegetarians - How well do their dietary patterns meet the Healthy People 2010 objectives? Arch Pediatr Adolesc Med. (2002) 156:431–7. 10.1001/archpedi.156.5.431 11980547

[34] Cifelli CJ Houchins JA Demmer E Fulgoni VL. Increasing plant based foods or dairy foods differentially affects nutrient intakes: dietary scenarios Using NHANES 2007-2010. Nutrients. (2016) 8:422. 10.3390/nu8070422 27409633 PMC4963898

[35] Elorinne AL Alfthan G Erlund I Kivimaki H Paju A Salminen I. Food and nutrient intake and nutritional status of Finnish vegans and non-vegetarians. PLoS ONE. (2016) 11:e0148235. 10.1371/journal.pone.0148235 26840251 PMC4739591

[36] Brenna JT. Efficiency of conversion of alpha-linolenic acid to long chain n-3 fatty acids in man. Curr Opin Clin Nutr Metab Care. (2002) 5:127–32. 10.1097/00075197-200203000-00002 11844977

[37] Greupner T Kutzner L Pagenkopf S Kohrs H Hahn A Schebb NH Schuchardt JP. Effects of a low and a high dietary LA/ALA ratio on long-chain PUFA concentrations in red blood cells. Food Funct. (2018) 9:4742–54. 10.1039/C8FO00735G 30101962

[38] Jackson SL King SM Zhao L Cogswell ME. Prevalence of excess sodium intake in the United States - NHANES, 2009-2012. MMWR Morb Mortal Wkly Rep. (2016) 64:1393–7. 10.15585/mmwr.mm6452a1 26741238

[39] Malta D Petersen KS Johnson C Trieu K Rae S Jefferson K. High sodium intake increases blood pressure and risk of kidney disease. From the Science of Salt: A regularly updated systematic review of salt and health outcomes (August 2016 to March 2017). J Clin Hypertens. (2018) 20:1654–65. 10.1111/jch.13408 30402970 PMC8030856

[40] Quader ZS Gillespie C Sliwa SA Ahuja JK Burdg JP Moshfegh A. Sodium intake among US school-aged children: National Health and Nutrition Examination Survey, 2011-2012. J Acad Nutr Diet. (2017) 117:39–47.e5. 10.1016/j.jand.2016.09.010 27818138 PMC5458522

[41] Drewnowski A Rehm CD. Sodium intakes of US children and adults from foods and beverages by location of origin and by specific food source. Nutrients. (2013) 5:1840–55. 10.3390/nu5061840 23760055 PMC3725480

[42] Grimes CA Wright JD Liu K Nowson CA Loria CM. Dietary sodium intake is associated with total fluid and sugar-sweetened beverage consumption in US children and adolescents aged 2-18 y: NHANES 2005-2008. Am J Clin Nutr. (2013) 98:189–96. 10.3945/ajcn.112.051508 23676421 PMC3683818

[43] O'Neil CE Nicklas TA Fulgoni VL 3rd Food sources of energy and nutrients of public health concern and nutrients to limit with a focus on milk and other dairy foods in children 2 to 18 years of age: National Health and Nutrition Examination Survey, 2011-2014. Nutrients. (2018) 10:1050 10.3390/nu10081050 PMC6116120 30096892

[44] Sabate J Lindsted KD Harris RD Sanchez A. Attained height of lacto-ovo vegetarian children and adolescents. Eur J Clin Nutr. (1991) 45:51–8. 1855500

[45] Gorczyca D Prescha A Szeremeta K Jankowski A. Iron status and dietary iron intake of vegetarian children from Poland. Ann Nutr Metab. (2013) 62:291–7. 10.1159/000348437 23712019

[46] Nathan I Hackett AF Kirby S. The dietary intake of a group of vegetarian children aged 7-11 years compared with matched omnivores. Br J Nutr. (1996) 75:533–44. 10.1079/BJN19960157 8672406

[47] Yen CE Yen CH Huang MC Cheng CH Huang YC. Dietary intake and nutritional status of vegetarian and omnivorous preschool children and their parents in Taiwan. Nutr Res. (2008) 28:430–6. 10.1016/j.nutres.2008.03.012 19083442

[48] Raiten DJ Namaste S Brabin B Combs G Jr L'Abbe MR Wasantwisut E. Executive summary–biomarkers of nutrition for development: building a consensus. Am J Clin Nutr. (2011) 94:633s−50s. 10.3945/ajcn.110.008227 21733880 PMC3142731

[49] Jun S Cowan AE Tooze JA Gahche JJ Dwyer JT Eicher-Miller HA Dietary supplement use among U.S. children by family income, food security level, and nutrition assistance program participation status in 2011-2014. Nutrients. (2018) 10:1212 10.3390/nu10091212 PMC6163871 30200511

