# Supplementary material for: Beyond Meat: A Comparison of the Dietary Intakes of Vegetarian and Non-vegetarian Adolescents
Source: Front Nutr. 2019 Jun 13;6:86. doi: 10.3389/fnut.2019.00086 (PMC6584749; doi:10.3389/fnut.2019.00086)
Supplement: Supplementary file 1 [file Table_1.DOCX]

**SUPPLEMENTARY TABLE**

| **FOOD GROUP** | **FOODS** |
| --- | --- |
| Breads, Grains, Pastas | Regular bread, flat bread, bagels/English muffins, breads/buns in sandwiches; pasta/noodles in dishes (with white sauce, red sauce, mac and cheese), ramen noodles; rice (white, brown, fried), rice in dishes, sushi/kimbap; pancakes; tortilla bread from burritos, tacos, quesadillas; pizza dough |
| Cereals | Ready-to-eat cereals, hot cereals, granola, cereal bars |
| Fresh fruits | Melons, apples/pears, peaches/plums/nectarines, oranges, grapes, berries, tropical fruits, banana |
| 100% fruit juices, canned, dried fruits | 100% real fruit juice, dried fruits, canned/stewed fruits and sauces |
| Non-starchy vegetables | Leafy green vegetables; salads and salad greens; snack vegetables; cabbage family; corn and peas; green and string beans; squash; eggplant; peppers; tomatoes; vegetables in spring rolls, vegetable dishes, vegetarian sushi, sandwiches, and pizza (vegetarian); avocado/guacamole |
| Starchy vegetables | Potatoes—fried/French fries, baked/boiled/canned, mashed; |
| Legumes | Beans/Legumes in mixed bean soups, lentil/split pea soups, haystack, hummus; edamame; soy nuts; beans in burritos, tacos, nachos; beans in bean dishes |
| Meats, processed meats, poultry | Meats eaten solely as steak/roast, meatloaf/meatballs, meat patties, meat links/franks/sausages; hotdogs; hamburger; chicken; meats in other sandwiches, burritos, tacos, quesadilla, pizza, spring rolls, and nachos; meats in rice, pasta/noodle, and bean dishes |
| Fish | Fish (all types); fish/seafood in sushi |
| Meat alternatives/soy products | Tofu, vegemeat patties, vegechicken, vegetarian fish, vegelinks/franks, tempeh, tofu in soups and dishes; vegemeats in dishes |
| Nuts and nut butters | Peanut butter in sandwich; nuts and nut butters; almond in almond milk |
| Eggs | Eggs; egg in breakfast burritos and breakfast sandwiches |
| Dairy cheese | Cheese eaten alone; cheese from pizza, sandwiches, burritos, tacos, quesadilla, and nachos; cream cheese; cottage cheese |
| Dairy milk | Regular, low-fat, and non-fat milk; milk in coffee blends, whey protein; milk in cream-based soups |
| Dairy substitutes | Soymilk, rice milk; milk substitutes in smoothies/shakes and coffee blends; non-dairy yogurt and ice cream |
| Dairy desserts | Dairy Ice cream; dairy yogurt; smoothies/shakes with dairy milk or whey |
| Water | Water |
| Sugar-sweetened beverages | Regular soda; diet soda; iced tea; sports drinks; fruit drinks |
| Coffee and tea | Black coffee, blended coffee, tea with artificial sweetener, tea with sugar |
| Pastries and chips | Popcorn, potato chips, corn/tortilla chips, other chips, other snacks, pastries, donuts, cakes/cookies |
